# Comparison of clinical efficacy between endoscope-assisted oblique lumbar interbody fusion and posterior lumbar interbody fusion in the treatment of lumbar degenerative diseases

**DOI:** 10.3389/fsurg.2026.1811413

**Published:** 2026-05-28

**Authors:** Haoran Han, Can Cao, Zekai Sun, Wenyi Li

**Affiliations:** 1 Department of Orthopedics, Hebei General Hospital, Shijiazhuang, Hebei, China; 2 Hebei Medical University, Shijiazhuang, Hebei, China

**Keywords:** lumbar degenerative disease, minimally invasive surgery, oblique lumbar interbody fusion, posterior lumbar interbody fusion, spinal endoscope

## Abstract

**Objective:**

To compare the clinical efficacy of endoscope-assisted oblique lumbar interbody fusion (Endo-OLIF) with posterior lumbar interbody fusion (PLIF) in the treatment of lumbar degenerative diseases.

**Methods:**

A retrospective analysis was conducted on 62 patients with lumbar degenerative diseases treated at Hebei General Hospital between January 2020 and December 2023. According to the surgical procedure, they were divided into an Endo-OLIF group (30 cases) and a PLIF group (32 cases). The two groups were compared in terms of baseline characteristics, operative time, intra-operative blood loss, post-operative drainage volume, length of hospital stay, visual analogue scale (VAS) scores, Oswestry Disability Index (ODI), fusion rate, cage subsidence rate, complication rate, and relevant imaging parameters (disc height, lumbar lordosis angle, fused-segment lordosis angle, and dural sac area).

**Results:**

There were no significant differences between the two groups in baseline characteristics such as sex, age, affected segment, comorbidities, or body-mass index (BMI) (*P* > 0.05). The Endo-OLIF group demonstrated significantly shorter operative time, less intra-operative blood loss, lower post-operative drainage, shorter hospital stay, and lower early post-operative VAS and ODI scores than the PLIF group (*P* < 0.05).However, VAS and ODI scores at 1 year post-operatively showed no significant inter-group difference (*P* > 0.05). Cage fusion and subsidence rates did not differ significantly between the two groups (*P* > 0.05). There was no statistically significant difference in the incidence of complications between the two groups (*P* > 0.05). Restoration of disc height, lumbar lordosis, and segmental lordosis was significantly better in the Endo-OLIF group (*P* < 0.05).

**Conclusions:**

The Endo-OLIF technique achieves direct and indirect spinal-canal decompression and restores segmental lumbar lordosis without disrupting the posterior osseous framework or the muscular-ligamentous complex, thereby expanding the surgical indications of conventional OLIF. Both procedures yield satisfactory clinical outcomes for lumbar degenerative diseases; however, the Endo-OLIF approach offers the advantages of minimal invasiveness, reduced blood loss, significantly lower early post-operative VAS scores, better restoration of lumbar physiological curvature, faster recovery, and a lower risk of neurological injury. Based on these limited but promising cases, this technique warrants broader clinical application and long-term follow-up.

## 1 Introduction

Lumbar degenerative disease is a common degenerative disorder associated with the natural aging process, and is recognized as one of the major causes of low back and leg pain, abnormal lower extremity sensation, and limited motor function in middle-aged and elderly populations (1–3). The main pathological characteristics include intervertebral disc degeneration and herniation, vertebral osteophyte formation, ligamentum flavum hypertrophy, and spinal and lateral recess stenosis. With a protracted course and recurrent symptoms, this condition severely reduces patients’ quality of life and accounts for a large proportion of clinical cases in spine surgery.

Posterior lumbar interbody fusion (PLIF) has long been regarded as the gold-standard procedure for degenerative lumbar disease, achieving satisfactory clinical outcomes via sufficient posterior spinal canal decompression. However, PLIF has been criticized for its long operative time, extensive surgical trauma, and relatively high incidence of postoperative complications (4). As an emerging minimally invasive surgical technique, oblique lumbar interbody fusion (OLIF) has attracted widespread attention from spine surgeons owing to its minimal invasiveness.

Traditional OLIF preserves the posterior bony structures and avoids disruption of the paraspinal muscles, facilitating rapid postoperative recovery (5–8). Nevertheless, due to its indirect decompression mechanism, traditional OLIF fails to achieve direct spinal canal decompression in cases with sequestrated disc herniation (8). Therefore, based on the direct decompression achieved through spinal endoscopy, we propose the endoscopic-assisted OLIF(Endo-OLIF), which combines spinal endoscopy with the OLIF technique. Under endoscopic visualization, the intervertebral space and spinal canal can be thoroughly explored, and herniated disc fragments can be removed, thereby achieving direct spinal canal decompression and expanding the clinical indications of OLIF.

Although Endo-OLIF enables direct decompression of the spinal canal, its decompression efficacy and clinical outcomes remain unclear compared with conventional open decompression surgery. Accordingly, this retrospective case-control study was performed to compare the clinical and radiological outcomes of Endo-OLIF vs. PLIF in the treatment of lumbar degenerative disease, aiming to provide evidence for clinical decision-making.

## 2 Material and methods

### 2.1 Study population

This study was a retrospective cohort study involving patients who underwent either Endo-OLIF or PLIF for lumbar degenerative diseases at our hospital between January 2020 and December 2023, and the retrospective study was approved by the Ethics Committee of Hebei General Hospital. Patient demographics, perioperative data, radiographic parameters, and clinical outcomes were retrospectively collected and reviewed.

### 2.2 Inclusion and exclusion criteria

#### 2.2.1 Inclusion criteria

Patients aged 18–85 years; those with low back and lower extremity symptoms lasting more than 3 months and unresponsive to conservative treatment; those diagnosed with lumbar degenerative diseases based on clinical symptoms and imaging examinations; those with complete follow-up data and a follow-up duration of more than 12 months.

#### 2.2.2 Exclusion criteria

Patients with a history of previous lumbar spine surgery; those with concurrent other spinal pathologies (e.g., infection, tumor, fracture); those with mental illness or cognitive impairment; those with incomplete follow-up data.

### 2.3 Surgical protocol

#### 2.3.1 Endo-OLIF surgery

After general anesthesia, the patient was placed in the right lateral decubitus position, and a left-sided approach was adopted. The surgical segment was localized under C-arm fluoroscopy and marked on the body surface (an oblique incision 2–3 cm ventral to the midpoint of the target intervertebral disc). The skin was incised along the planned route, followed by blunt dissection of the external oblique muscle, internal oblique muscle, transverse abdominal muscle, and peritoneal tissue. The retroperitoneal tissues and psoas major muscle were retracted anteriorly and posteriorly, respectively, and the surgical segment was accessed through the natural space between the abdominal aorta and psoas major muscle. Care was taken to avoid injury to the ureter, sympathetic chain, spermatic vein or ovarian vein, renal artery branches, and other vital structures during the dissection process.

In each of the target segments, 2.5 mm Kirschner wires were inserted as positioning pins and the positioning of the target intervertebral space was re-performed with fluoroscopy. Using a special right-angle deep retractor, pull the peritoneum and ureter towards the ventral side, and pull the lumbar fascia towards the dorsal side, exposing the intervertebral space of the target segment. The “fence” technique with 3–4 Kirschner wires was used to shield important structures from the operative field. The annulus fibrosus was incised, and the nucleus pulposus tissue in the intervertebral disc as well as the upper and lower cartilaginous endplates were removed.

Insert the spinal endoscope into the intervertebral space, and the direction of the endoscope was flexibly adjusted. With the assistance of curved forceps, nucleus pulposus forceps, radiofrequency probes, and a steerable burr, the spinal canal could be safely entered for exploration and removal of the corresponding tissues invading the spinal canal. This allowed for direct decompression of the spinal canal; under endoscopic visualization, the expansion of the dural sac and the mobility of the nerve roots were observed, enabling direct evaluation of the immediate decompression effect.

**Figure 1:**
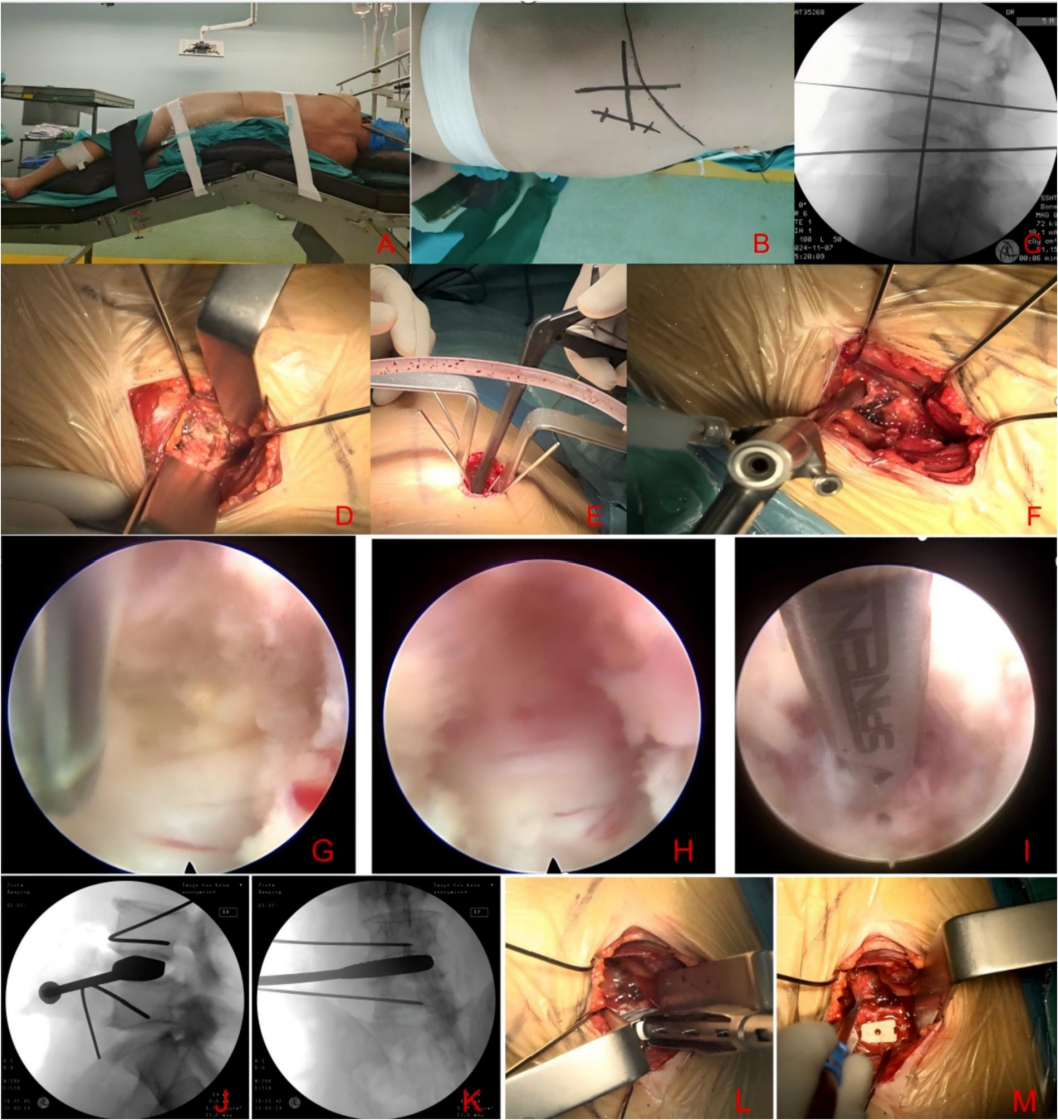
Surgical position **(A)** identification of the target intervertebral space **(B,C)**. Exposure of the target intervertebral space and Kirschner wire “fence” technique **(D,E)**. Insertion of the spinal endoscopy system **(F)** Direct decompression of the spinal canal under spinal endoscopy **(G–I)**. Insertion of the interbody fusion cage **(J–M)**.

After decompression was completed, the intervertebral space was appropriately distracted, and trial distractors of different sizes were tested. A suitable-sized cage filled with allogeneic bone was implanted into the corresponding intervertebral space. Intraoperative fluoroscopy confirmed that the position and size of the cage were satisfactory, and then the wound was irrigated before internal fixation was performed. According to the different internal fixation methods, it can be divided into lateral fixation and posterior fixation. The advantage of lateral fixation over posterior fixation is that the fixation can be performed under direct vision and there is no need to adjust the patient's position during the operation; however, its mechanical strength is inferior to that of posterior screw internal fixation. Therefore, when the lumbar spine has poor stability and rigid fixation is required, posterior screw fixation remains the first choice (Figure 1).

#### 2.3.2 PLIF surgery

**Figure 2:**
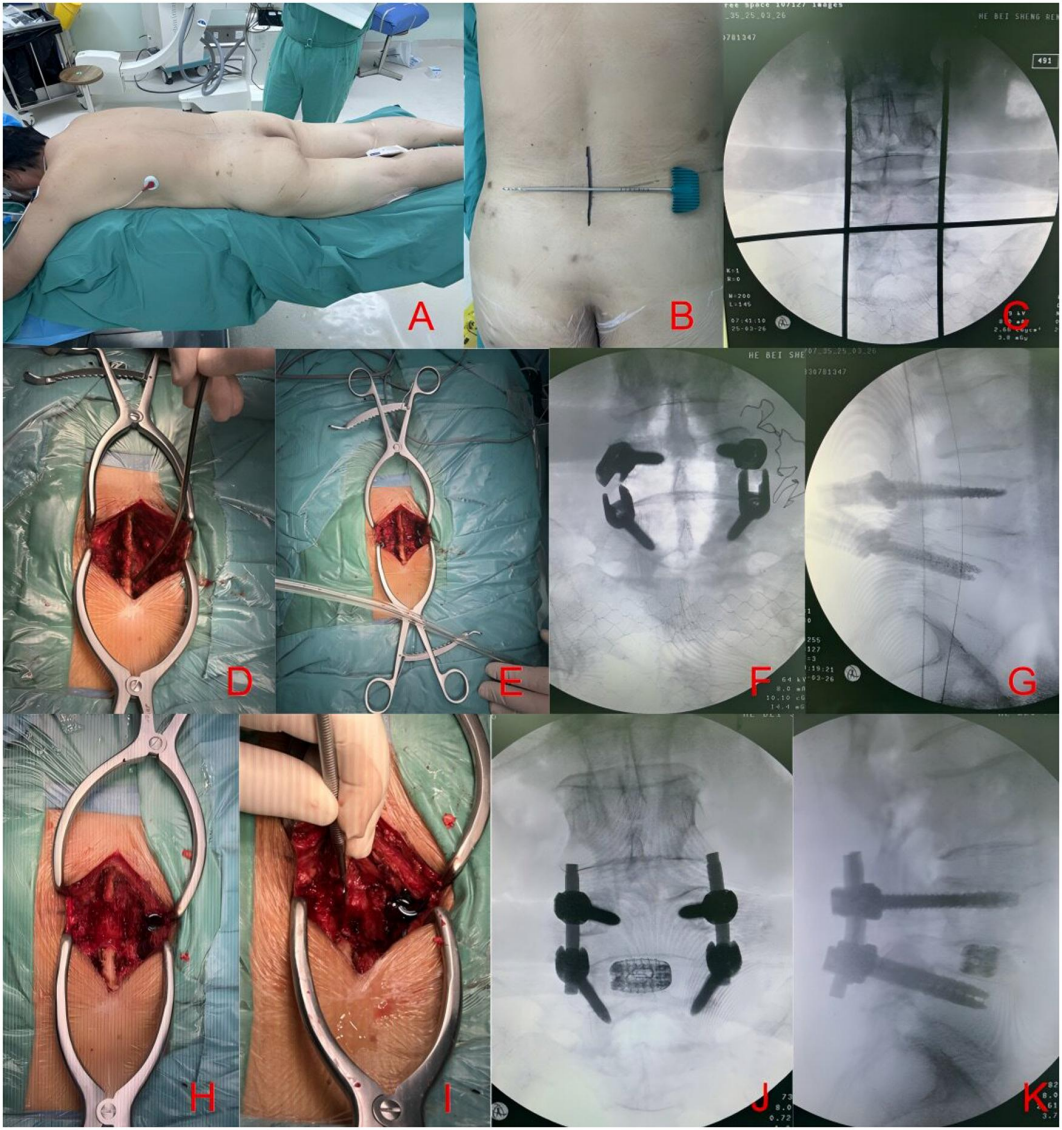
Surgical position **(A)** identification of the target intervertebral space **(B,C)**. The paraspinal muscles were dissected bilaterally along the spinous processes to fully expose the spinous processes and laminae **(D)**Pedicle screw insertion**(E–G)**. Posterior laminectomy and dural sac exposure **(H)** The nucleus pulposus of the intervertebral space was excised **(I)**. Insertion of the interbody fusion cage **(J,K)**.

Following general anesthesia, the patient was placed in the prone position. The affected segment was identified under C-arm fluoroscopy. A midline longitudinal incision was made along the posterior lumbar spine. The skin and superficial fascia were dissected layer by layer, and the paraspinal muscles were sufficiently stripped off the spinous processes to expose the bony structures including the spinous processes, laminae, and facet joints posterior to the lumbar vertebrae. Pedicle screws were inserted into the pedicles of the upper and lower vertebral bodies at the affected segment, and C-arm fluoroscopy confirmed satisfactory screw position. The spinous processes, laminae, partial facet joints, and hypertrophic ligamentum flavum at the affected level were resected to relieve the compression on the dural sac and nerve roots. A nerve retractor was used to retract the dural sac and nerve roots, and the annulus fibrosus at the affected level was incised. The degenerated nucleus pulposus was thoroughly removed using a curette and nucleus pulposus forceps, and the upper and lower cartilaginous endplates were prepared. An appropriately sized interbody fusion cage filled with autologous bone graft was inserted into the intervertebral space. The connecting rod was connected to the screws, and the set screws were tightened for compression and fixation. Fluoroscopy was repeated to confirm the satisfactory position of the fusion cage and pedicle screws. A drainage tube was placed, and the incision was closed layer by layer (Figure 2).

### 2.4 Postoperative management

The operative time, intraoperative blood loss, postoperative drainage volume, hospital stay and incidence of complications were recorded for all patients. Postoperative management was identical between the two groups: same postoperative mobilization schedule, unified analgesic regimen, consistent lumbar brace usage, and standardized physiotherapy by the same team. A drainage tube was routinely placed in patients of both groups after surgery, with close observation of the drainage volume and the nature of drainage fluid. The drainage tube was removed when the drainage volume was less than 50 ml per 24 h. Patients were allowed to ambulate with brace protection postoperatively, with each ambulation session limited to no more than 15 min.

### 2.5 Clinical efficacy assessment

Before and after surgery, the Visual Analog Scale (VAS; scoring range: 0–10 points, with higher scores indicating more severe pain) and Oswestry Disability Index (ODI; higher scores indicating more severe functional impairment) were used to evaluate the patients’ clinical symptoms.

### 2.6 Imaging assessment

**Figure 3:**
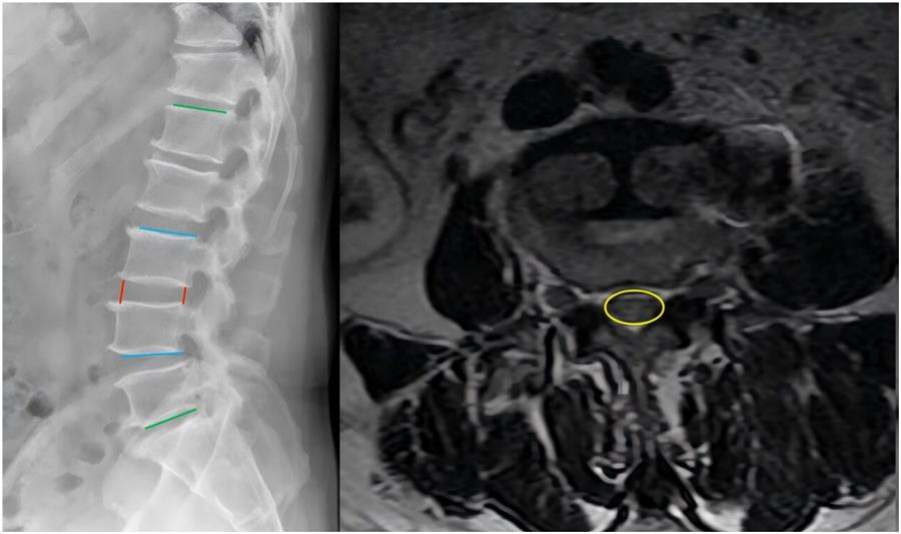
Measurement of imaging parameters. Disc height (DH) was defined as the average of the anterior and posterior heights of the affected segment (red solid lines). Lumbar lordosis angle was measured as the angle between the superior endplate of L1 and the inferior endplate of L5 (green solid lines). Fused segment lordosis angle was determined as the angle between the superior and inferior endplates of the adjacent vertebrae in the fused segment (blue solid lines). Dural sac cross-sectional area (DSCA) was manually measured at the narrowest portion of the dural sac on T2-weighted MRI at the corresponding level of the affected segment, both preoperatively and postoperatively (The area delineated by the yellow solid line). Bone graft fusion was considered successful if continuous trabecular bone formation was observed between the cage and adjacent vertebrae on postoperative lumbar CT; otherwise, it was defined as non-union. The fusion rate was calculated as the ratio of successfully fused cases to the total number of surgical cases.

For the assessment of long-term clinical outcomes, all patients underwent regular follow-up, and imaging examinations were conducted at the final follow-up. The imaging parameters were measured at preoperation, 3 days postoperation, and final follow-up, including disc height (DH), lumbar lordosis angle, fused segment lordosis angle, and dural sac cross-sectional area (DSCA) (Figure 3). DH was defined as the average of the anterior and posterior heights of the affected segment. Lumbar lordosis angle was measured as the angle between the superior endplate of L1 and the inferior endplate of L5. Fused segment lordosis angle was determined as the angle between the superior and inferior endplates of the adjacent vertebrae in the fused segment. DSCA was manually measured at the narrowest portion of the dural sac on T2-weighted MRI at the corresponding level of the affected segment.

At the 1-year postoperative follow-up, the lumbar CT examination was performed to evaluate the fusion status. Successful fusion was determined by the presence of continuous trabecular bone bridging between the cage and adjacent vertebrae. While fusion failure was diagnosed in the absence of such bone formation. The fusion rate was computed as the ratio of successful fusion cases to the total number of all patients. In addition, cage subsidence was observed, defined as inferior cage migration into the vertebral body exceeding 2 mm.

### 2.7 Statistical analysis

All statistical analyses were conducted using SPSS 26.0 software (IBM Corp., Armonk, NY, USA). Continuous data were presented as mean ± standard deviation (SD) and compared via independent-samples *t*-test. Categorical data were analyzed using the chi-square test. Statistical significance was set at *P* < 0.05.

## 3 Results

### 3.1 Baseline characteristics

A total of 62 patients were included in this study. Based on the surgical protocols, 30 patients were divided into the Endo-OLIF group and the 32 patients into PLIF group. The Endo-OLIF group consisted of 17 males and 13 females, with a mean age of (67.50 ± 8.53) years and a mean BMI of (27.18 ± 3.90) kg/m^2^. In this group, 21 patients underwent single-level Endo-OLIF and 9 patients underwent multi-level Endo-OLIF. The PLIF group consisted of 12 males and 20 females, with a mean age of (65.09 ± 5.38) years and a mean BMI of (27.54 ± 3.36) kg/m^2^. Of these, 25 patients underwent single-level surgery and 7 underwent multi-level surgery. No significant differences were observed in age, gender, BMI, or surgical levels between the Endo-OLIF group and the PLIF group (*P* > 0.05) (Table 1).

**Table 1:** Baseline characteristics of patients in Two groups.

Variables	Endo-OLIF group	PLIF group	*P*
Age (years)	67.50 ± 8.53	65.09 ± 5.38	0.187
Gender (Male/Female)	17/13	12/20	0.131
BMI (kg/m^2^)	27.18 ± 3.90	27.54 ± 3.36	0.697
Surgical Segments			0.386
L1–2	1	0	
L2–3	0	1	
L3–4	3	4	
L4–5	17	19	
L5–S1	0	1	
L3–4 & L4–5	9	5	
L4–5 & L5–S1	0	2	

### 3.2 Surgical trauma assessment

For the Endo-OLIF group, the mean operation time was 162.33 ± 38.61 min, intraoperative blood loss was 50.00 ± 28.64 ml, postoperative drainage output was 58.53 ± 53.99 ml, and hospital stay was 11.83 ± 3.73 days. For the PLIF group, the corresponding values were 201.22 ± 52.19 min, 354.06 ± 182.58 ml, 293.59 ± 118.78 ml, and 16.94 ± 5.68 days, respectively. Statistical analysis showed significant differences in these indicators between the two groups (*P* < 0.05), with the Endo-OLIF group exhibiting shorter operation time, reduced intraoperative blood loss, lower postoperative drainage, and shorter hospital stay compared with the PLIF group (Table 2).

**Table 2:** Comparison of surgical trauma-related outcomes between the endo-OLIF and PLIF groups.

Indicators	Endo-OLIF group	PLIF group	*P*
Operation Time (min)	162.33 ± 38.61	201.22 ± 52.19	0.002
Intraoperative Blood Loss (ml)	50.00 ± 28.64	354.06 ± 182.58	<0.001
Postoperative Drainage Volume (ml)	58.53 ± 53.99	293.59 ± 118.78	<0.001
Length of Hospital Stay (d)	11.83 ± 3.73	16.94 ± 5.68	<0.001

### 3.3 Clinical efficacy assessment

No significant differences were observed in preoperative VAS scores for lumbocrural pain or ODI scores between the two groups (*P* > 0.05). Postoperatively, both groups exhibited a significant reduction in VAS and ODI scores compared with the preoperative period, reflecting a substantial alleviation of clinical symptoms. However, the Endo-OLIF group had significantly lower early postoperative VAS and ODI scores than the PLIF group (*P* < 0.05). After a follow-up of more than 1 year, there were no significant differences in VAS or ODI scores between the two groups (Table 3).

**Table 3:** Comparisons of VAS and ODI scores at different time points between two groups.

Scores	Time points	Endo-OLIF group	PLIF group	*P*
VAS	Preoperation	6.40 ± 1.00	6.44 ± 0.98	0.882
	3 Days Postoperation	3.00 ± 1.00	3.66 ± 0.97	0.023
	1 Month Postoperation	2.43 ± 0.62	3.06 ± 0.80	0.001
	1 Year Postoperation	2.40 ± 1.00	2.47 ± 0.72	0.756
ODI(%)	Preoperation	60.42 ± 8.28	56.81 ± 10.87	0.148
	1 Month Postoperation	22.33 ± 3.75	27.44 ± 7.63	0.001
	1 Year Postoperation	13.82 ± 2.41	14.24 ± 3.22	0.570

A Repeated-measures ANOVA was performed for VAS scores and ODI scores. Mauchly's test indicated violation of sphericity (*P* < 0.05) in both analyses; therefore, Greenhouse-Geisser correction was applied. For VAS scores, the time main effect was significant (*P* < 0.001), indicating that both procedures relieved low back pain over time. The group main effect was significant (*P* = 0.042), with the Endo-OLIF group showing better overall pain relief. A significant Time × Group interaction was observed (*P* = 0.036), demonstrating different improvement trajectories: the Endo-OLIF group achieved earlier and more sustained pain improvement, whereas the PLIF group had a slower recovery. For ODI scores, the time main effect was significant (*P* < 0.001), indicating that both procedures improved lumbar function over time. The group main effect was not significant (*P* = 0.607), with the two groups showing comparable overall functional recovery. A significant Time × Group interaction was detected (*P* = 0.002), indicating distinct functional recovery patterns: the Endo-OLIF group exhibited earlier and more rapid functional improvement, while the PLIF group showed a more gradual recovery. These findings suggest that Endo-OLIF provides faster and more effective pain and functional relief than PLIF.

### 3.4 Complications assessment

Postoperatively, complications occurred in 7 of 30 patients (23.33%) in the Endo-OLIF group. Five patients experienced thigh numbness and pain: 2 cases resolved spontaneously within 1 week, and 3 cases underwent additional minimally invasive spinal endoscopic surgery at 1 week, with marked symptomatic relief. Two patients had cage subsidence but remained asymptomatic during follow-up. All patients achieved satisfactory clinical outcomes.

In the PLIF group, complications were noted in 8 of 32 patients (25%): 2 cases of dural tear, which resolved after 1 month of conservative treatment; 2 cases of lower-extremity deep vein thrombosis (DVT) treated with routine heparin anticoagulation; and 4 cases of cage subsidence. One patient with cage subsidence underwent revision surgery, while the remaining 3 were asymptomatic and managed with long-term follow-up. No significant between-group difference was observed in the complication rate (*P* = 0.878).

### 3.5 Imageological assessment

No significant differences were observed in disc height (DH), lumbar lordosis angle, fused segment lordosis angle, or dural sac cross-sectional area (DSCA) between the two groups preoperatively. Postoperatively, all these parameters were significantly improved in both groups compared with the preoperative measurements. The Endo-OLIF group demonstrated superior improvements in DH, lumbar lordosis angle, and fused segment lordosis angle at 3 days postoperatively and 1 year postoperatively compared with the PLIF group (all *P* < 0.05). For DSCA, the preoperative and postoperative values were (41.37 ± 15.06) mm^2^ and (69.87 ± 20.94) mm^2^ in the Endo-OLIF group, and (41.18 ± 26.24) mm^2^ and (91.18 ± 28.48) mm^2^ in the PLIF group, respectively. Significant improvements in DSCA were noted postoperatively in both groups. However, as the PLIF procedure achieves open spinal canal decompression via the posterior approach, the PLIF group had a larger postoperative DSCA than the Endo-OLIF group (*P* < 0.05) (Table 4).

**Table 4:** Comparisons of imaging parameters at different time points between Two groups.

Imaging parameters	Time Points	Endo-OLIF group	PLIF group	*P*
Disc height (mm)	Preoperation	9.46 ± 1.34	8.68 ± 2.27	0.069
	Postoperation	12.77 ± 1.62	11.38 ± 1.99	0.001
	1 Year Postoperation	13.05 ± 1.41	11.21 ± 1.69	<0.001
Lumbar lordosis angle (°)	Preoperation	37.31 ± 12.22	36.34 ± 10.72	0.727
	Postoperation	43.77 ± 10.76	38.34 ± 10.32	0.047
	1 Year Postoperation	43.93 ± 10.73	37.94 ± 10.09	0.027
Fused segment lordosis angle (°)	Preoperation	12.92 ± 6.35	13.08 ± 6.52	0.916
	Postoperation	19.74 ± 4.95	16.18 ± 7.22	0.014
	1 Year Postoperation	20.56 ± 4.40	16.86 ± 5.80	0.002
Dural sac cross-sectional area (mm^2^)	Preoperation	41.37 ± 15.06	41.18 ± 26.24	0.184
	Postoperation	69.87 ± 20.94	91.18 ± 28.48	<0.001
Bone graft fusion rate (%)	1 Year Postoperation	96.7% (29/30)	93.8% (30/32)	0.593

A Repeated-measures ANOVA was performed for lumbar lordosis angle, disc height, and fused segment lordosis angle preoperatively, postoperatively, and at 1-year follow-up, as well as for dural sac cross-sectional area preoperatively and postoperatively. Mauchly's test indicated a violation of sphericity (*P* < 0.05) in all analyses; therefore, Greenhouse-Geisser correction was applied. For lumbar lordosis angle, the main effect of time was significant (*P* < 0.001), while the main effect of group was not significant (*P* = 0.607), and a significant Time × Group interaction was observed (*P* = 0.002), indicating distinct improvement trajectories over time. For disc height, significant main effects of time (*P* < 0.001) and group *(P* < 0.001) were detected, accompanied by a significant Time × Group interaction (*P* = 0.022). For fused segment lordosis angle, the main effect of time was significant (*P* < 0.001), while the main effect of group was not significant (*P* = 0.059), and a significant Time × Group interaction was found (*P* < 0.001). For dural sac cross-sectional area, significant main effects of time (*P* < 0.001) and group (*P* = 0.039) were observed, with a significant Time × Group interaction (*P* < 0.001). These results demonstrated that both groups achieved favorable radiographic correction postoperatively, while the Endo-OLIF group exhibited earlier and more favorable recovery in radiological parameters.

### 3.6 Assessment of bone graft fusion rate

Regardingthe bone graft fusion rate, 29 cases achieved successful fusion at 1 year postoperatively in the Endo-OLIF group, yielding a fusion rate of 96.7% (29/30). In the PLIF group, 30 cases attained successful fusion at 1 year postoperatively, with a fusion rate of 93.8% (30/32). No significant intergroup difference was observed in the postoperative fusion rate in the follow-up cohort of this study (*P* > 0.05) (Table 4).

### 3.7 Subgroup analysis of internal fixation method in endo-OLIF

In the present study, two fixation modalities were used in the Endo-OLIF group: lateral vertebral screw fixation (*n* = 19) and posterior pedicle screw fixation (*n* = 11). The subgroup analysis was performed to evaluate the clinical outcomes in different fixation strategies of Endo-OLIF.

The operation time, intraoperative blood loss, postoperative drainage volume, and length of hospital stay were slightly higher in the Endo-OLIF plus posterior pedicle screw fixation subgroup than in the Endo-OLIF plus lateral fixation subgroup; however, no statistically significant differences were observed between the two subgroups in these perioperative parameters (Table 5). Both groups exhibited significant improvements in postoperative VAS scores and Oswestry Disability Index (ODI) compared with preoperative values, with no significant intergroup differences (Table 6).

**Table 5:** Comparison of surgical trauma-related outcomes between different endo-OLIF subgroups.

Indicators	Endo-OLIF with lateral fixation	Endo-OLIF with posterior pedicle screw fixation	*P*
Operation time (min)	152.89 ± 38.61	178.64 ± 55.27	0.164
Intraoperative blood loss (ml)	45.79 ± 28.15	57.27 ± 29.35	0.298
Postoperative drainage volume (ml)	55.11 ± 31.85	64.45 ± 81.03	0.656
Length of hospital stay (d)	11.26 ± 4.11	12.82 ± 2.89	0.279

**Table 6:** Comparison of VAS and ODI scores at different time points between different endo-OLIF subgroups.

Scores	Time points	Endo-OLIF with lateral fixation	Endo-OLIF with posterior pedicle screw fixation	*P*
VAS	Preoperation	6.21 ± 1.08	6.73 ± 0.79	0.178
	3 Days Postoperation	2.95 ± 1.18	3.09 ± 1.38	0.764
	1 Month Postoperation	2.47 ± 0.61	2.36 ± 0.67	0.651
	1 Year Postoperation	2.58 ± 1.12	2.09 ± 0.70	0.205
ODI(%)	Preoperation	58.72 ± 8.38	63.36 ± 7.58	0.142
	1 Month Postoperation	22.98 ± 4.01	21.21 ± 3.11	0.218
	1 Year Postoperation	14.09 ± 2.56	13.35 ± 1.99	0.428

Regarding radiographic outcomes, the disc height, lumbar lordosis angle, fused segment lordosis angle, and dural sac cross-sectional area were significantly improved postoperatively in both subgroups relative to preoperative measurements, and no significant intergroup differences were found in any radiographic parameters. The bone graft fusion rate was 94.74% in the Endo-OLIF plus lateral fixation subgroup and 100% in the Endo-OLIF plus posterior pedicle screw fixation subgroup, with no significant intergroup difference in fusion rate (Table 7). In summary, subgroup analysis demonstrated that both lateral fixation and posterior pedicle screw fixation in Endo-OLIF can achieve satisfactory clinical and radiographic outcomes.

**Table 7:** Comparison of imaging parameters at different time points between different endo-OLIF subgroups.

Imaging parameters	Time points	Endo-OLIF with lateral fixation	Endo-OLIF with posterior pedicle screw fixation	*P*
Disc height (mm)	Preoperation	9.34 ± 1.24	9.72 ± 1.58	0.420
	Postoperation	13.00 ± 1.55	12.23 ± 1.72	0.178
	Final Follow-up	13.26 ± 1.44	12.61 ± 1.32	0.191
Lumbar lordosis angle (°)	Preoperation	36.00 ± 13.65	39.73 ± 9.37	0.430
	Postoperation	42.63 ± 11.76	45.73 ± 8.97	0.458
	Final Follow-up	43.68 ± 8.38	44.36 ± 8.14	0.871
Fused segment lordosis angle (°)	Preoperation	12.11 ± 5.88	14.75 ± 7.25	0.236
	Postoperation	19.44 ± 4.69	20.42 ± 5.66	0.578
	Final Follow-up	20.19 ± 4.12	21.42 ± 5.05	0.427
Dural sac cross-sectional area (mm^2^)	Preoperation	41.81 ± 15.62	40.27 ± 14.23	0.779
	Postoperation	72.18 ± 23.46	64.67 ± 13.07	0.307
Bone graft fusion rate (%)	Final Follow-up	94.74% (18/19)	100% (11/11)	0.439

## 4 Discussion

Since first introduced by Mayer (5) in 1997, the OLIF technique has developed rapidly; conventional OLIF achieves indirect spinal canal decompression via oblique lateral implantation of a large interbody fusion cage without resecting posterior vertebral structures. Although previous studies (9–12) have confirmed that the indirect decompression efficacy of conventional OLIF is comparable to that of direct decompression with other surgical procedures, conventional OLIF still exhibits several inherent limitations with regard to indirect decompression.

Due to the indirect decompression mechanism, conventional OLIF is not indicated for spinal stenosis caused by sequestrated nucleus pulposus, fat deposition, space-occupying lesions, or bony stenosis secondary to posterior longitudinal ligament calcification. Thus, its surgical indications are more limited than those of PLIF. To address this, our institution combined spinal endoscopy with OLIF, which retains conventional OLIF's advantages, enables safe spinal canal access via high-definition endoscopy (avoiding dural sac/nerve root injury), and achieves direct decompression by precisely resecting herniated disc fragments and calcified ligaments, thus broadening its clinical applicability.

### 4.1 Comparison of clinical outcomes between the two surgical procedures

Both Endo-OLIF and PLIF achieve satisfactory outcomes in lumbar degenerative diseases (9, 13, 14), with significantly improved postoperative VAS and ODI scores in both groups and no significant difference at 1 year postoperatively. However, early VAS and ODI improvements were more prominent in the Endo-OLIF group, attributed to its less invasive oblique approach with a smaller incision and no damage to the posterior musculoligamentous complex, resulting in less incision pain.

The oblique approach of Endo-OLIF also allows a larger interbody fusion cage while preserving posterior soft tissues, providing better lumbar stability than PLIF, which was an important factor in reducing postoperative low back pain. Tang (15) reported 33.54% (106/316) postoperative low back pain in PLIF patients, noting that multifidus muscle condition and early functional exercise are key determinants. In contrast to PLIF that disrupts posterior structures and uses smaller cages, Endo-OLIF avoids posterior musculoligamentous injury, thereby reducing both the incidence and severity of postoperative pain. Internal fixation significantly affects lumbar stability. In this study, two fixation methods were combined with Endo-OLIF, including lateral screw fixation in 19 cases and posterior pedicle screw fixation in 11 cases. Lateral screw fixation offers multiple advantages, including no need for patient repositioning, fewer incisions, shorter operation time, less blood loss, and lower fluoroscopy frequency. However, it provides relatively inferior spinal stability. Therefore, posterior pedicle screw fixation was preferred in later procedures. As demonstrated by Hao (16) posterior pedicle screw fixation yields optimal lumbar stability with the lowest segmental motion and implant stress. This strategy also reduces the risk of cage subsidence and provides better long-term outcomes, despite longer operation time and more blood loss related to intraoperative repositioning.

### 4.2 Comparison of surgical trauma between the two surgical procedures

Endo-OLIF accesses the affected segment via the natural space between the psoas major and major abdominal vessels, using blunt tissue dissection and endoscopic electrocoagulation hemostasis during endplate preparation, resulting in significantly less intraoperative blood loss than PLIF. In this study, the mean operative time of the Endo-OLIF group was 162.33 ± 38.61 min, significantly shorter than 201.22 ± 52.19 min in the PLIF group, which partly contributed to its lower blood loss.Compared with PLIF, Endo-OLIF has a shorter mean surgical incision (5 cm), which not only reduces postoperative pain but also lowers the risk of surgical site infection. In the present study, the Endo-OLIF group had a significantly lower mean postoperative drainage volume (58.53 ± 53.99 ml) than the PLIF group (293.59 ± 118.78 ml), and the larger drainage volume in the PLIF group not only prolonged catheter drainage and ambulation time but also correspondingly increased the risk of drainage tube-related infection. Among the subjects enrolled in this study, patients in the Endo-OLIF group generally resumed ambulation on the first postoperative day, whereas those undergoing PLIF typically got out of bed at 3–4 days. In summary, Endo-OLIF achieves minimal invasiveness, faster recovery and less trauma without compromising therapeutic efficacy, in contrast to PLIF.

### 4.3 Comparison of imaging parameters between the two surgical procedures

The Endo-OLIF cage is wider with a built-in lordotic angle and can span the entire endplates, which theoretically contributes to the restoration of intervertebral height, lumbar lordosis, and sagittal balance. In this study, postoperative intervertebral height, lumbar lordosis, fused segmental lordosis, and dural sac area were significantly improved in both groups vs. preoperative values. On the third day after the operation and at the one-year postoperative follow-up, the improvement in intervertebral height, lumbar lordosis angle, and lordosis angle of the fusion segment in the Endo-OLIF group was significantly better than that in the control group (*P* < 0.05), which was in line with theoretical expectations.

There was a statistically significant difference in dural sac cross-sectional area between the two groups, and the PLIF group exhibited a larger dural sac area postoperatively. This can be attributed to the fact that PLIF achieves thorough decompression of the spinal canal by resecting the posterior compressive tissues. Although the decompression range of Endo-OLIF was narrower than PLIF, the postoperative clinical efficacy were comparable between the two groups. Adequate neural decompression, not dural sac area alone, determines clinical efficacy (17, 18). Endo-OLIF enables precise neural decompression without damaging posterior structures, providing better spinal stability and superior clinical outcomes.The wider OLIF cage theoretically allows higher fusion rates due to larger bone graft volume, but fusion rates were comparable between groups. This is because OLIF used mostly allogeneic bone, while PLIF used autologous bone (eliminating immune rejection), leading to similar fusion results.

Restoring lumbar sagittal balance reduces postoperative low back pain and adjacent segment disease. We prefer posterior pedicle screw fixation for its superior rigidity, better restoration of physiological lumbar curvature, and lower cage subsidence risk; Zhao W (19) confirmed OLIF combined with posterior fixation is superior to stand-alone OLIF in restoring lordosis.

In summary, Endo-OLIF better restores intervertebral height, lumbar lordosis, and fused segmental lordosis than PLIF, while ensuring efficacy and facilitating lumbar sagittal balance restoration.

### 4.4 Comparison of complications between the two surgical procedures

No ureteral or major vascular complications occurred in the Endo-OLIF group. Asymptomatic cage subsidence was observed in 2 cases, both associated with low preoperative BMD and inadequate anti-osteoporosis therapy. The relationship between BMD and cage subsidence remains controversial (20–23). For patients older than 60 years with BMD T-score <−1.0, we recommend supplementary posterior pedicle screw fixation to enhance stability and reduce subsidence. Song C (24) confirmed higher cage and endplate stress in osteoporotic spines, with posterior fixation providing optimal biomechanical stability. Cement-augmented screws may improve fixation strength (25), but carry a high leakage rate and may complicate revisions (26). For osteoporotic patients, routine anti-osteoporosis medication is advisable.Excessive endplate preparation increases cage subsidence and migration (27, 28). Endo-OLIF allows precise endplate preparation under direct visualization, and its wider cage distributes load more evenly, further reducing such risks.

Common OLIF complications include lower extremity pain, numbness, and hip flexion weakness, mostly reversible nerve traction injuries. In this study, 5 patients developed these symptoms; 2 recovered spontaneously, and 3 improved after endoscopic revision, mainly due to severe degeneration and prolonged operation time. The overall complication rate of Endo-OLIF was 23.33%, slightly higher than previously reported (15%–20%, mean 19%) (20, 29), likely due to the small sample size. In the PLIF group, complications included 2 dural tears, 2 deep vein thromboses, and 4 cage subsidences, with an overall rate of 21.87%. PLIF carries inherent risks of iatrogenic neural injury from extensive posterior dissection. There was no significant between-group difference in complication rate, suggesting that both procedures provide satisfactory safety.

### 4.5 Indications and contraindications of endo-OLIF

Conventional OLIF indications include mild-to-moderate lumbar spinal stenosis, degenerative scoliosis, segmental instability, Grade Ⅰ–Ⅱ spondylolisthesis, post-fusion adjacent segment disease, prior lumbar revision, discogenic low back pain, and intervertebral infection. Its core mechanism is restoring intervertebral height via lateral wide cage implantation, distracting the posterior longitudinal ligament and ligamentum flavum to indirectly expand the spinal canal without resecting posterior structures. However, it shows suboptimal efficacy for stenosis from sequestrated/prolapsed nucleus pulposus, fat deposition, or ligamentum flavum calcification-related bony stenosis. With advances in spinal endoscopic equipment and techniques, Endo-OLIF combination has been proposed (30–32). Based on conventional indirect decompression, this modality enables endoscopic resection of sequestrated nucleus pulposus, posterior osteophyte drilling, and direct nerve/dural sac decompression. Integrating both decompression methods, it overcomes conventional OLIF limitations, expanding indications to: (a) Lumbar instability with sequestrated disc herniation; (b) Severe stenosis with space-occupying lesions; (c) Posterior vertebral osteophytes; (d) Conventional OLIF revision.

Nevertheless, Endo-OLIF has relative and absolute contraindications: (a) Nucleus pulposus migrated dorsal to nerve roots and dural sac; (b) Stenosis from severe ligamentum flavum hypertrophy; (c) Severe lumbar spondylolisthesis (Grade Ⅲ+); (d) Congenital osseous spinal canal atresia; (e) Left-sided paracentral disc herniation; (f). Active abdominal infection or tuberculosis.

### 4.6 Limitations of the study

This study has several limitations. First, as a single-center retrospective study, Endo-OLIF is an emerging surgical technique, resulting in a relatively small sample size and short follow-up duration. Second, preoperative bone mineral density (BMD) was not standardized across both cohorts, which may impact the interpretation of implant stability. Third, and most importantly, this study represents a holistic pre- and post-surgical assessment. We did not stratify patients based on detailed degenerative imaging markers, including MODC, the Pfirrmann Classification, the Schizas Classification (stenosis), or even the Giutallier Classification. Consequently, the results reflect an overall clinical trend rather than the specific efficacy within a particular morphological subtype of lumbar degeneration. Fourth, this procedure has a long learning curve and high technical difficulty, requiring the surgeon to master spinal endoscopy skills proficiently on the basis of conventional OLIF experience.To further evaluate the efficacy of Endo-OLIF for the treatment of lumbar degenerative diseases, multi-center prospective studies and relevant investigations based on imaging-derived stratified data should be performed.

## 5 Conclusion

The Endo-OLIF technique achieves direct and indirect spinal-canal decompression and restores segmental lumbar lordosis without disrupting the posterior osseous framework or the muscular-ligamentous complex, thereby expanding the surgical indications of conventional OLIF. Both procedures yield satisfactory clinical outcomes for lumbar degenerative diseases; however, the Endo-OLIF approach offers the advantages of minimal invasiveness, reduced blood loss, significantly lower early post-operative VAS scores, better restoration of lumbar physiological curvature, faster recovery, and a lower risk of neurological injury. Based on these limited but promising cases, this technique warrants broader clinical application and long-term follow-up.

## Data Availability

The raw data supporting the conclusions of this article will be made available by the authors, without undue reservation.
